# Iontophoresis-Assisted Corneal Collagen Cross-Linking with Epithelial Debridement: Preliminary Results

**DOI:** 10.1155/2016/3720517

**Published:** 2016-07-28

**Authors:** Paolo Vinciguerra, Vito Romano, Pietro Rosetta, Emanuela F. Legrottaglie, Magdalena Kubrak-Kisza, Claudio Azzolini, Riccardo Vinciguerra

**Affiliations:** ^1^Humanitas Clinical and Research Center, Via Manzoni 56, 20089 Rozzano, Italy; ^2^Humanitas University, Rozzano, 20089 Milan, Italy; ^3^Department of Corneal and External Eye Diseases, St. Paul's Eye Unit, Royal Liverpool University Hospital, Liverpool L7 8XP, UK; ^4^Department and Clinic of Ophthalmology, Wrocław Medical University, 50-556 Wrocław, Poland; ^5^Department of Surgical Sciences, Division of Ophthalmology, University of Insubria, 21100 Varese, Italy; ^6^Department of Ophthalmology, Humanitas Mater Domini, Via Gerenzano 2, 21053 Castellanza, Italy

## Abstract

*Purpose*. To report the early outcomes of iontophoresis-assisted corneal collagen cross-linking procedure with epithelial debridement (I-SCXL).* Methods*. Twenty eyes of twenty patients with progressive keratoconus were included in this prospective clinical study. Best spectacle corrected visual acuity (BSCVA), sphere and cylinder refraction, corneal topography, Scheimpflug tomography, aberrometry, anterior segment optical coherence tomography (AS-OCT), and endothelial cell count were assessed at baseline and at 1, 3, and 6 months of follow-up. The parameters considered to establish keratoconus progression were always proven with differential maps as change in curvature in the cone area of at least 1 diopter obtained with an instantaneous map.* Results*. Functional parameters showed a significant improvement (*p* < 0.05) of BSCVA after 3 and 6 months of follow-up. Morphological parameters indicated stabilization of the corneal ectasia during the follow-up; however, a positive trend was noted with a mean flattening of 1.73 D. Minimum pachymetry values showed thinning that remained constant after the treatment. The demarcation line was clearly visible in all patients, reaching a depth of 308.2 ± 37.74 *μ*m. None of the patients had continuous progression of keratoconus or had to repeat cross-linking procedures. Endothelial cell counts did not change significantly (*p* > 0.05). *Conclusion*. The early results indicate that the I-SCXL may be able to reduce the treatment time and improve the riboflavin diffusion.

## 1. Introduction

Keratoconus (KC) is a common, progressive disorder of the cornea that results in corneal thinning and an increase in curvature. One of the key features of KC is a loss of corneal mechanical stability, leading to a reduction in stiffness of the cornea by up to 60% compared to the healthy state [1]. In particular, keratoconic corneas are characterized by a different distribution and expression of collagen fibrils and an alteration in the interfibrillar distance, which causes corneal instability [2]. Currently, standard collagen cross-linking (S-CXL) using ultraviolet A (UVA) irradiation combined with the photosensitiser riboflavin has been used to halt the progression of KC by “stiffening” the cornea [3–6].

The standard procedure entails the removal of the corneal epithelium to facilitate diffusion of riboflavin throughout the corneal stroma; however, the diffusion is not well standardised and also time-dependent. Preliminary preclinical results have shown that transepithelial CXL (TE-CXL) with iontophoresis increases the concentration of riboflavin inside the stroma compared to other TE-CXL techniques and also produces histological changes [7–11] reducing the amount of time required for the treatment. We have also reported the clinical data that show the efficacy of TE-CXL using iontophoresis [12].

It has been reported, however, that epithelial debridement followed by iontophoresis induces higher concentration of riboflavin inside corneal stroma and in the aqueous humour compared to the transepithelial iontophoresis approach [13].

The aim of this paper is to report the preliminary results of iontophoresis-assisted corneal collagen cross-linking with epithelial debridement (I-SCXL).

## 2. Materials and Methods

In this prospective, single centered, pilot clinical study, twenty eyes of 20 patients that underwent I-SCXL at the Eye Clinic, Humanitas Clinical and Research Center (Rozzano, Milan, Italy), were included. The inclusion criteria for the treatment of CXL were documentation of the progression of keratoconus, patients over the age of 9 years, and signed informed consent. The preoperative progression of keratoconus was demonstrated by at least two optical pachymetries with Scheimpflug camera and corneal differential topographies obtained after a minimum of three months. The parameters considered to establish keratoconus progression were always proven with differential maps, as change in curvature in the cone area of at least 1 diopter (D) obtained with instantaneous map or as thinning of more than 20 *μ*m in minimal pachymetry.

Exclusion criteria were a history of herpetic keratitis, dry eye, severe corneal infection, and concomitant ocular or systemic autoimmune disease.

Other exclusion criteria were a previously diagnosed pregnancy or breastfeeding, the presence of central or paracentral opacities, low compliance, and the use of rigid contact lenses less than 4 weeks before the baseline evaluation.

The study received Institutional Review Board approval from the ethical committee of Istituto Clinico Humanitas and was conducted accordingly to the ethical standards set in the 1964 Declaration of Helsinki, as revised in 2000. All patients provided informed consent.

In the preoperative and postoperative course (1, 3, and 6 months), the following parameters were assessed: best spectacle corrected visual acuity (BSCVA), slit lamp biomicroscopy, Goldmann tonometry, dilated fundoscopy, corneal topography and corneal aberrometry for the evaluation of low- and high-order aberrations (Costruzione Strumenti Oftalmici (CSO), Florence, Italy), optical tomography and pachymetry with Pentacam (Oculus Inc., Lynnwood, WA), anterior segment optical coherence tomography (AS-OCT), and endothelial biomicroscopy (Konan Specular Microscope, Konan Medical Inc, Hyogo, Japan).

### 2.1. Corneal Iontophoretic Cross-Linking Procedure with Epithelial Removal

I-SCXL was performed as a day-surgery procedure. It was conducted under topical anesthesia with 2 applications of 4% lidocaine drops and 0.2% oxybuprocaine hydrochloride under sterile conditions. Before the procedure, pain medication was administered (Ketorolac 10 mg 1 pill) and 2% pilocarpine drops were instilled in the eye to be treated to reduce the amount of ultraviolet light reaching the retina [2].

After the lid speculum was applied, the corneal epithelium was abraded in a central, 9-mm diameter area with the aid of an Amoils brush (Amoils Brush Ephthential Scrubber; Vision Technology Co., Seoul, Korea). Subsequently, the iontophoresis device for corneal application was placed on the cornea using an annular suction ring. The corneal iontophoresis electrode was filled with riboflavin solution (Ricrolin+, Sooft, Montegiorgio, FM, Italy) and the device was then connected to a constant current generator (I-ON XL, Sooft, Montegiorgio, FM, Italy) set at 1 mA for 5 minutes (the total dose of 5 mA/5 minutes is monitored by the generator). Afterwards, the cornea was irradiated at a working distance of 45 mm with UV lamp of 10 mW (UV-X 2000; IROC Innocross AG, Switzerland) for 9 minutes. A calibrated ultraviolet A meter (LaserMate-Q; Laser 2000, Wessling, Germany) was used before treatment to check the irradiance at a 1.0 cm distance. The total treatment time is 15 minutes.

At the end of surgery, a soft therapeutic contact lens was applied. Postoperative pain management protocol entailed Ketorolac 10 mg 1 pill every 8 hours. An ophthalmic gel containing 0.15% sodium hyaluronate, 1% xanthan gum, and 0.3% netilmicin (Xanternet; SIFI SpA, Catania, Italy) was prescribed 4 times a day until no epithelial damage was observed (epithelial integrity was evaluated with fluorescein staining every day postoperatively). After removal of contact lens, dexamethasone 21-phosphate 0.15% drops (Etacortilen, SIFI, Lavinaio, Italy) twice daily for 10 days and 0.15% sodium hyaluronate drops (BluYal, Sooft, Montegiorgio, Italy) 6 times daily for 45 days were prescribed. In addition, all patients received oral amino acid supplements (Aminoftal, Sooft, Montegiorgio, Italy) for 7 days, this because it has been demonstrated that reepithelialization is improved when an increase of serum and tear film amino acids is obtained through oral administration [14].

All preoperative and postoperative functional and morphological tests were performed in an identical manner to a previously published clinical study [5, 12, 15], and postoperative complications were recorded.

Statistical analysis was performed using the SPSS statistics software (IBM Corp., Armonk, New York) version 20.0. Data are described by mean and standard deviation. All data samples were first analysed using the Shapiro-Wilk test. Student's* t*-test for paired data was applied to assess the significance of differences between preoperative and postoperative data using the same level of significance (*p* < 0.05) in all cases.

## 3. Results

Twenty eyes of 20 patients (16 males and 4 females) were evaluated. The mean age of patients was 30.8 ± 6.6 years with a range of 20–45 years. The mean preoperative *K*_max_ was 53.63 ± 5.60 diopters with range of 44–66.2. The mean preoperative thickness was 473.6 ± 36.50 *μ*m with a range of 417–523 *μ*m. All patients, except one that did not present at last visit, were evaluated at each follow-up (1-3-6) till 6 months postoperatively. Clinical features of the patients and their preoperative data are listed in Table 1. Comparative analysis at 1, 3, and 6 months of follow-up showed the following findings reported in Table 1.

### 3.1. Functional Analysis

#### 3.1.1. Visual Acuity

Best Spectacle Corrected Visual Acuity (BSCVA) showed a significant improvement at 3 and 6 months (*p* = 0.01; *p* = 0.002, resp.) starting from a mean of 20/30 (0.69 ± 0.17 decimals). At 1 month after treatment the BSCVA showed an improvement, although it is not significant, compared to baseline.

#### 3.1.2. Refractive Results

After I-SCXL sphere and cylinder refraction did not show significant change through the follow-up (*p* > 0.05).

#### 3.1.3. Aberrometric Results

Aberrometric results showed a significant worsening in high-order aberration (HOA), COMA, and spherical aberration at month 1 (*p* < 0.05). Subsequently, COMA and HOA showed a nonsignificant improvement, compared to baseline (*p* > 0.05).

### 3.2. Structural Analysis

#### 3.2.1. Topographic Results

Topographic results are showed in Table 1. The morphological indices indicate stabilization of keratoconus. The analysis of the topographic results showed significant improvement of corneal symmetry index (SI) at last follow-up; all the other indices were stable for the entire period. *K*_max_ displayed a mean reduction of −1.73 D of *K*_max_; however this result did not reach statistical significance (*p* = 0.3). Minimum pachymetry values showed significant thinning at month 1 that remained constant afterwards (*p* < 0.05).

**Figure 1:**
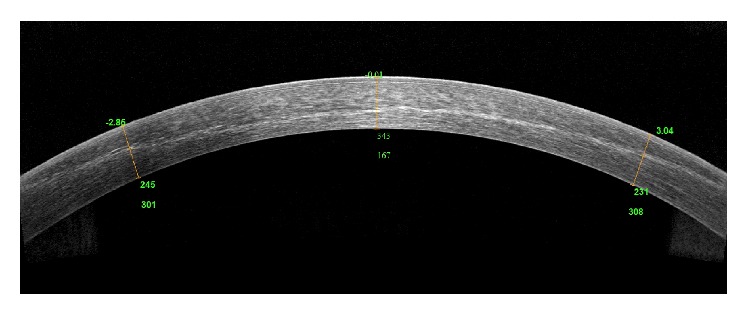
Showing clear and deep demarcation line after I-SCXL.

The evaluation of anterior segment OCT showed a clearly visible demarcation line in all the patients at 1 month with a mean reached depth of 308.2 ± 37.74 *μ*m (Figure 1).

Preoperative mean endothelial cell count was 2280 ± 224 cell/mm^2^ and did not show a significant difference during all follow-up (*p* > 0.05). None of the patients developed infection or haze.

## 4. Discussion

The original Dresden corneal collagen cross-linking protocol, which includes epithelial removal, was introduced as a 60 minute process to treat progressive keratoconus [4]. However, the passive impregnation of the corneal stroma with riboflavin induces a concentration inside the stroma as a function of depth and is time-dependent. Iontophoresis is an active method of forcing riboflavin through the tissue, which accelerates the process while maintaining optimal efficacy. Various studies have previously showed that transepithelial iontophoresis is able to induce a significantly higher concentration of riboflavin inside the stroma, although lower than standard CXL with epithelial removal (S-CXL) [4, 7, 9, 16]. It has been reported that iontophoresis impregnation with epithelial debridement induces a higher concentration of riboflavin inside corneal stroma and in the aqueous humour compared to the transepithelial iontophoresis approach [13]. This evidence suggested that I-SXCL might be a suitable alternative to S-CXL to reduce the treatment time while maintaining optimal efficacy.

To the best of our knowledge, this is the first prospective clinical study in which preoperative and postoperative refractive, topographic, tomographic, and aberrometric outcomes have been analysed in eyes with progressive keratoconus treated with a commercial ocular iontophoresis device after epithelial removal.

The six-month functional results showed significant improvement in BSCVA after 3 months and this was confirmed at last follow-up. This finding is an important improvement compared to I-SCXL, as in the standard protocol, vision normally improves only after 6–12 months [3, 6, 15].

Comatic, spherical, and higher-order aberrations remained stable during follow-up, compared to baseline, after an initial worsening. Once more, this positive functional improvement was concomitant, with no significant worsening of morphological and functional parameters at the 1-month follow-up visit (typical of standard CXL) [3, 6, 15].

Maximum keratometry (*K*_max_) showed a mean flattening of 1.7 D at last follow-up; however this change was not significant.

Another finding that supports the concept that the iontophoresis may be effective to improve the riboflavin diffusion after epithelial removal was the deep and strong demarcation lines (DL) in all the evaluated patients. Indeed, it is known that, after epi-off CXL procedures (either standard or accelerated), a stromal demarcation line is normally observed inside the corneal stroma at 1 month postoperatively [17–23]. Moreover, DL is considered an indirect measurement of CXL penetration within the stroma [24].

Previous reports showed that the pulsed corneal cross-linking protocol induced a significantly deeper stromal demarcation line when compared to the 4 minutes of highly accelerated continuous CXL protocol [25, 26]. Moramarco et al. reported the mean depth of demarcation line at 149 *μ*m in patients treated with accelerated CXL (continuous UVA light exposure at 30 mW/cm^2^ for 4 minutes and energy dose of 7.2 J/cm) and 213 *μ*m in patients treated with pulsed UVA light (pulsed UVA light exposure at 30 mW/cm^2^ for 8 minutes, 1 second on/1 second off, and energy dose of 7.2 J/cm) [25]. Peyman et al. found the stromal demarcation line depth at 201 *μ*m in pulsed group versus 159 *μ*m in continuous group [26], while Kymionis et al. reported a deeper demarcation line (approximately between 300 and 337 *μ*m) in conventional CXL treatment compared to accelerated high-intensity 10-minute CXL [27, 28]. It has been recently reported that iontophoresis-assisted transepithelial CXL creates a demarcation line distinguishable at a mean depth of 242–247 *μ*m [29]. In our study the demarcation line was clearly visible at AS-OCT in all the patients after 1-month follow-up. The depth was a mean 308 *μ*m with minimum of 268 *μ*m and maximum of 396 *μ*m. It is still not clear whether the stromal demarcation line depends on the mode of treatment dose or on the riboflavin diffusion; more research will help to better understand the pathophysiology of this finding.

These data show the capacity of iontophoresis to improve the soaking time, in terms of reducing the treatment time and increasing the diffusion of riboflavin, as indicated by demarcation line. Larger study populations will be necessary to reach this conclusion. Additionally, I-SCXL proved to be a safe technique, as demonstrated by the stability of endothelial cell count and no complications. The principal limits of our study are the relatively low number of patients, the lack of comparison with S-CXL, and the number of months of follow-up. The number of patients included was chosen as we already published three articles, one in Ophthalmology in 2009 [5], one in Journal of Refractive Surgery in 2014 [12], and one under revision in the Journal of Refractive Surgery in which we always included 20 patients for the evaluation of standard CXL and the latter two of transepithelial iontophoresis.

Further studies are required to understand if I-SCXL can improve the long-term results of the gold standard CXL procedure.

In conclusion, I-SCXL has the potential to improve the standard CXL in order to halt the progression of the ectatic disease reducing treatment time and improving the soaking time.

## Figures and Tables

**Table 1:** Clinical outcome of standard corneal collagen cross-linking using iontophoresis (I-SCXL).

Outcome	Baseline	One month	*p*	Three months	*p*	Six months	*p*
BSCVA	0.69 ± 0.17	0.73 ± 0.18	0.33	0.81 ± 0.21	0.01	0.82 ± 0.17	0.002
Sphere	−1.13 ± 2.04	−0.37 ± 2.06	0.04	−0.86 ± 1.67	0.6	−0.54 ± 1.49	0.1
Cylinder	−3.25 ± 1.26	−3.76 ± 1.62	0.1	−3.51 ± 1.66	0.4	−2.90 ± 1.36	0.1
AX	105 ± 36.24	107.5 ± 29.49	0.8	95.5 ± 34.9	0.3	110.3 ± 26.57	0.5
*K*_max_	53.63 ± 5.60	53.67 ± 6.38	0.9	53.51 ± 5.63	1	51.9 ± 4.89	0.3
SAI	6.17 ± 3.65	6.91 ± 3.63	0.06	6.47 ± 3.43	0.3	4.26 ± 3.50	0.1
SI	7.35 ± 2.83	7.45 ± 2.88	0.3	7.36 ± 2.98	0.9	6.95 ± 2.85	0.02
ISV	84.35 ± 29.75	91.2 ± 32.9	0.06	87.5 ± 31.47	0.2	79.88 ± 33.32	0.8
IVA	0.98 ± 0.39	1.05 ± 0.45	0.1	0.93 ± 0.37	0.6	0.93 ± 0.42	0.7
KI	1.21 ± 0.08	1.23 ± 0.99	0.004	1.22 ± 0.09	0.1	1.20 ± 0.09	0.5
CKI	1.03 ± 0.05	1.05 ± 0.05	0.01	1.04 ± 0.05	0.4	1.02 ± 0.04	0.6
IHA	31.62 ± 27.54	26.39 ± 22.25	0.3	29.51 ± 20.09	0.6	21.3 ± 13.54	0.1
IHD	0.08 ± 0.03	0.08 ± 0.03	0.5	0.08 ± 0.03	0.8	0.10 ± 0.11	0.3
*R*_min_	6.36 ± 0.62	6.30 ± 0.77	0.4	6.36 ± 0.65	0.1	6.45 ± 0.65	0.2
ThCT	473.6 ± 36.50	441.8 ± 41.72	<0.001	441.35 ± 49.32	<0.001	452.7 ± 53.10	0.0002
HOA	0.32 ± 0.15	0.36 ± 0.15	0.008	0.31 ± 0.13	0.57	0.30 ± 0.13	0.85
COMA	0.51 ± 0.33	0.64 ± 0.37	0.0002	0.56 ± 0.36	0.09	0.46 ± 0.32	0.89
Absph	−0.03 ± 0.06	0.007 ± 0.08	0.02	−0.013 ± 0.08	0.1	−0.03 ± 0.07	0.64

BSCVA: best spectacle corrected visual acuity; sphere: sphere refraction; cylinder: cylinder; AX: axis of the cylinder; *K*_max_: maximum keratometry; SAI: surface asymmetry index; SI: symmetry index; ISV: index of surface variance; IVA: index of vertical asymmetry; KI: keratoconus index; CKI: central keratoconus index; IHA: index of height asymmetry; IHD: index of height decentration; *R*_min_: minimum radius of curvature; ThCT: minimum pachymetry; HOA: high-order aberration; COMA: comatic aberration; Absph: spherical aberration.
